# Supplementary material for: Assessment Heartworm Disease in the Canary Islands (Spain): Risk of Transmission in a Hyperendemic Area by Ecological Niche Modeling and Its Future Projection
Source: Animals (Basel). 2023 Oct 18;13(20):3251. doi: 10.3390/ani13203251 (PMC10603702; doi:10.3390/ani13203251)
Supplement: Supplementary file 1 [file animals-13-03251-s001.zip › Additional file 13.pdf]

Regularization multiplier	10
Feature classes	q (cuadratic)
Sets of predictors	Set 1

Additional file 13.
