# Supplementary figures and images for: Assessment Heartworm Disease in the Canary Islands (Spain): Risk of Transmission in a Hyperendemic Area by Ecological Niche Modeling and Its Future Projection
Source: Animals (Basel). 2023 Oct 18;13(20):3251. doi: 10.3390/ani13203251 (PMC10603702; doi:10.3390/ani13203251)

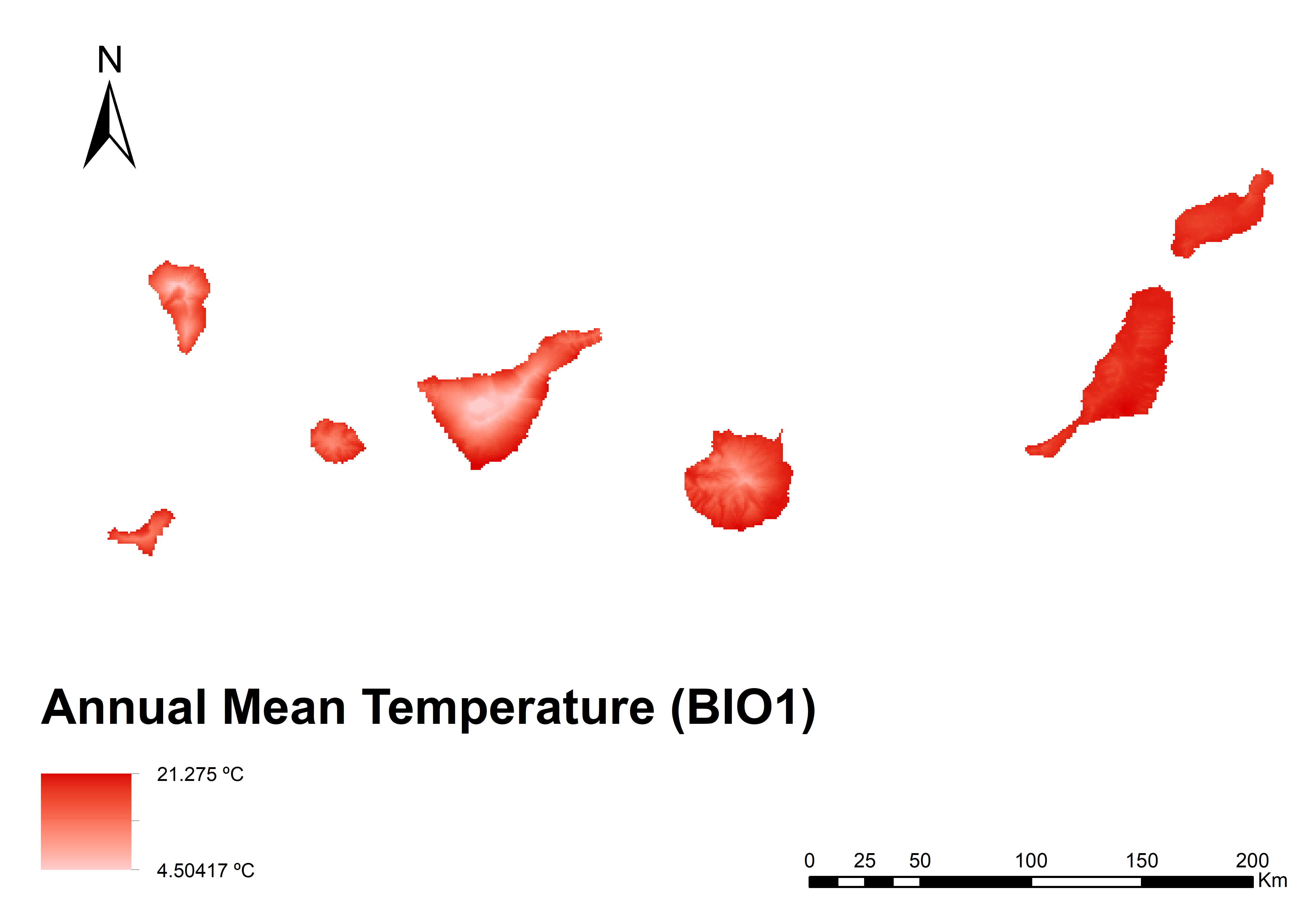

Supplement: Supplementary file 1 [file animals-13-03251-s001.zip › Additional file 1.tif]

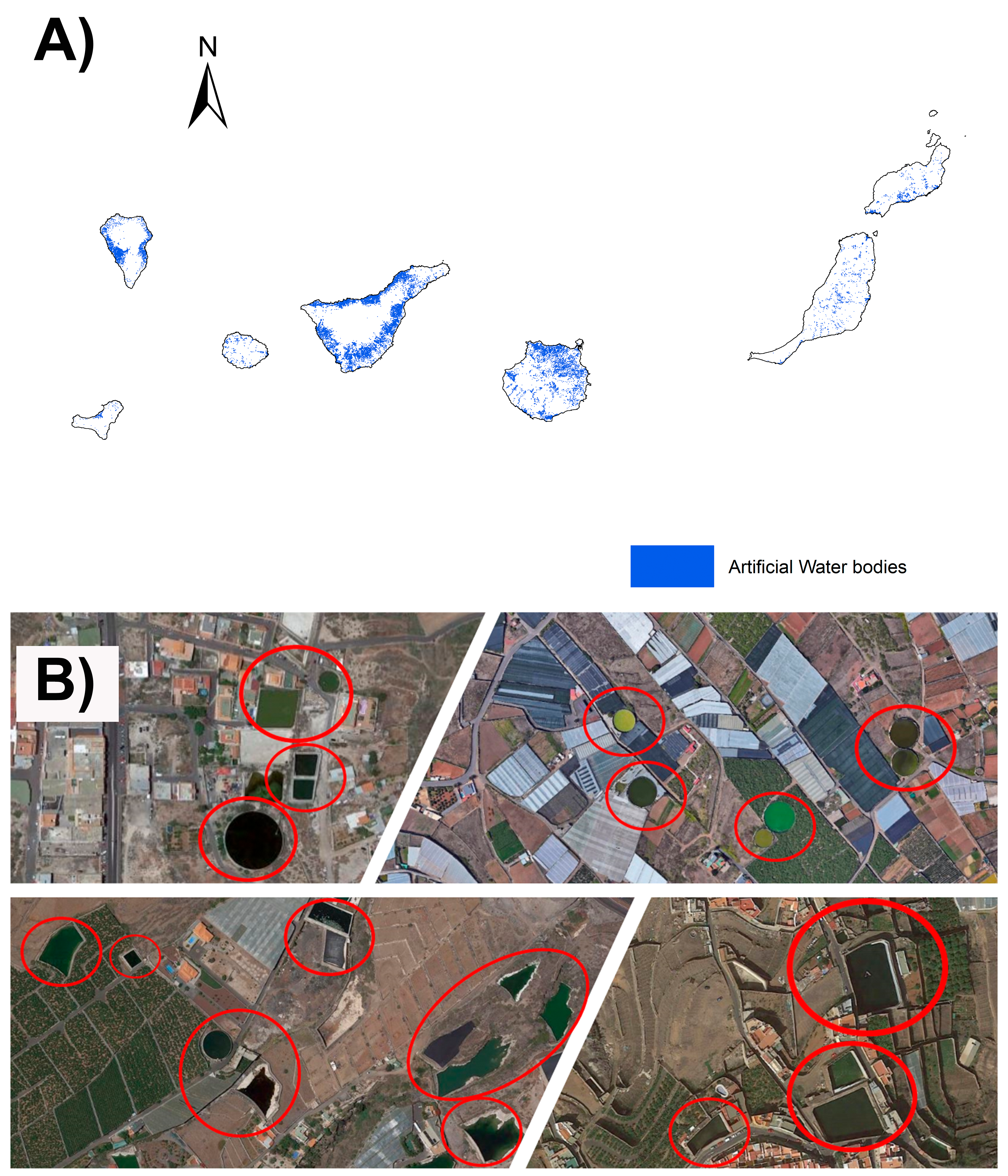

Supplement: Supplementary file 1 [file animals-13-03251-s001.zip › Additional file 10.tif]

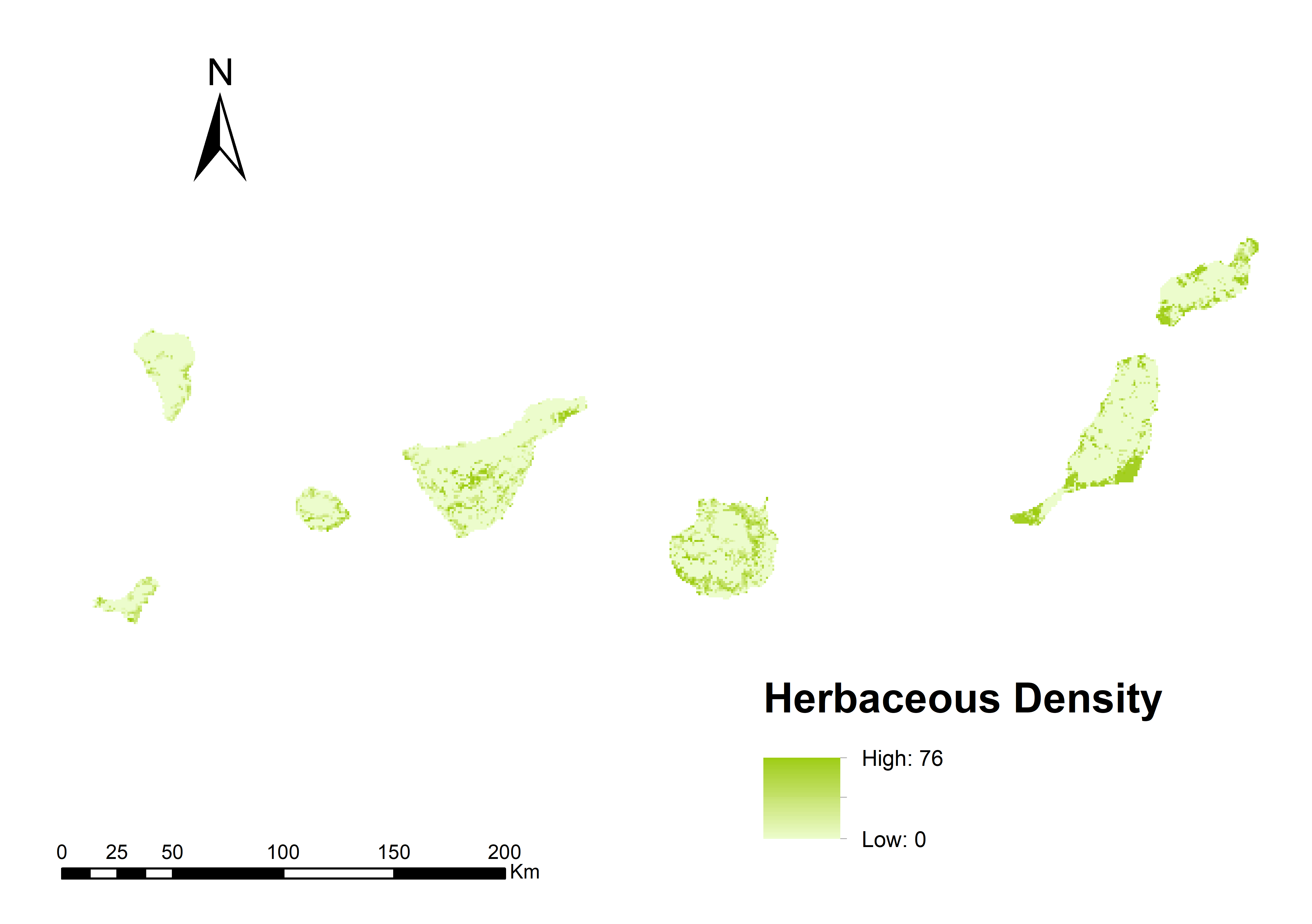

Supplement: Supplementary file 1 [file animals-13-03251-s001.zip › Additional file 11.tif]

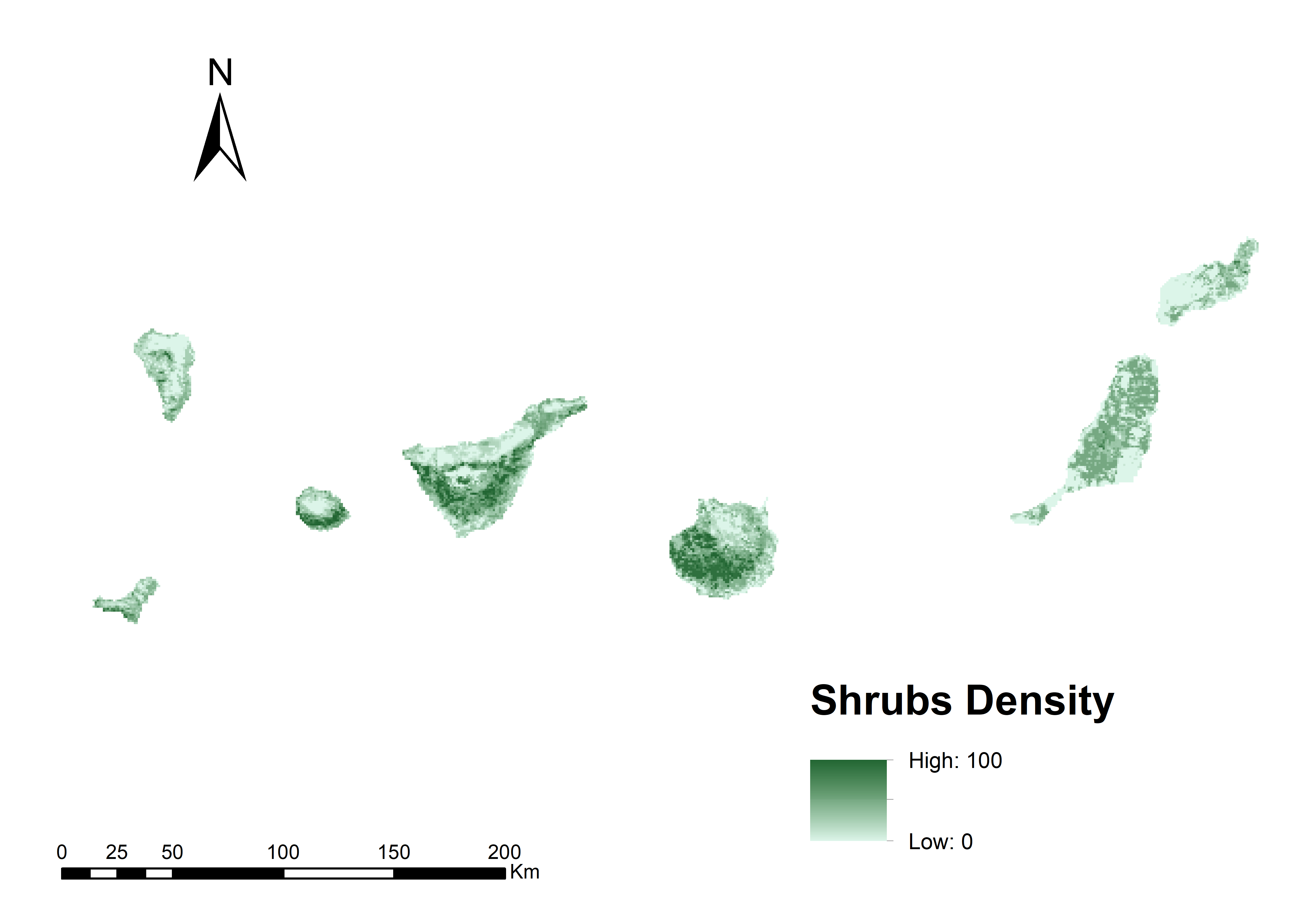

Supplement: Supplementary file 1 [file animals-13-03251-s001.zip › Additional file 12.tif]

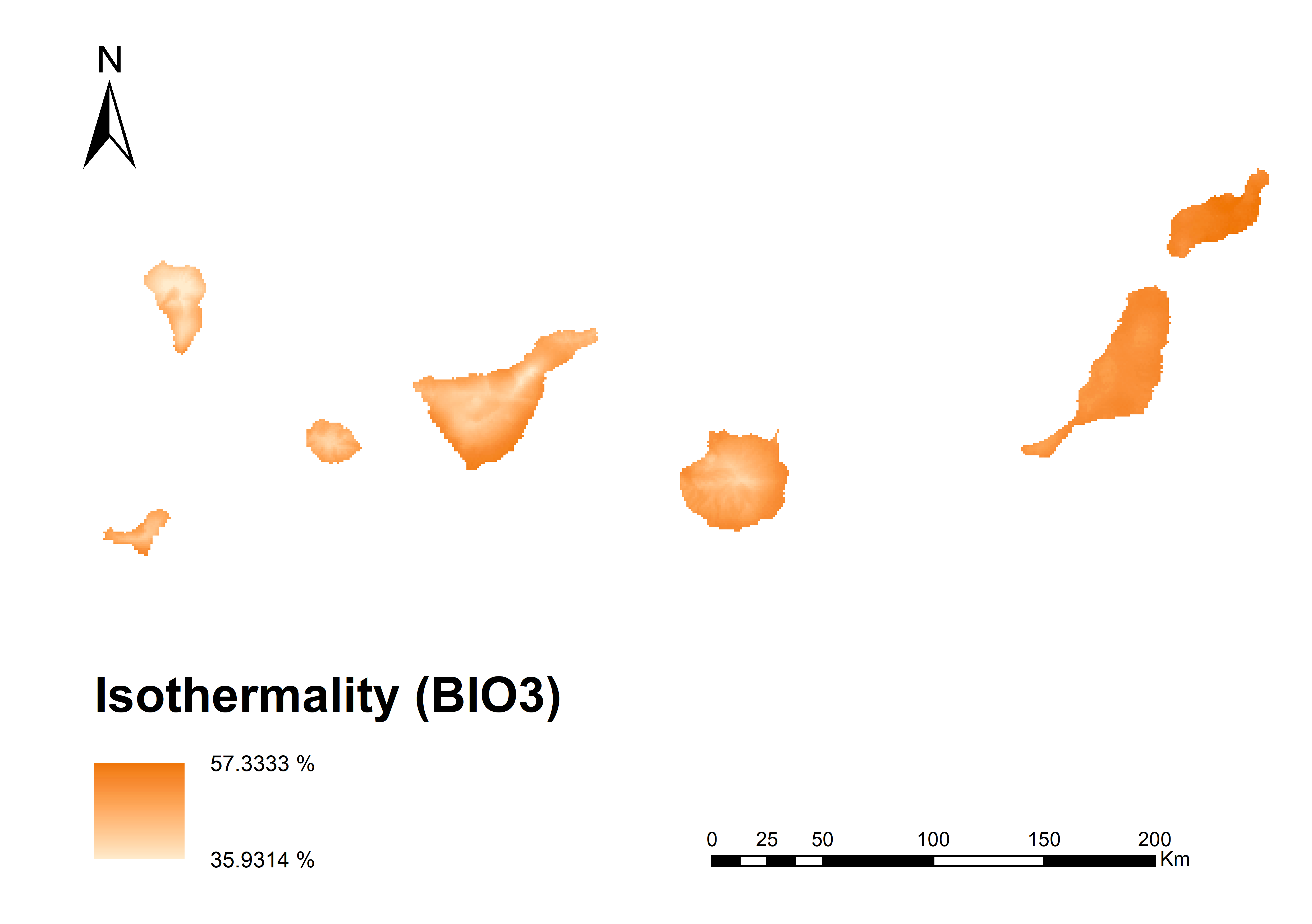

Supplement: Supplementary file 1 [file animals-13-03251-s001.zip › Additional file 2.tif]

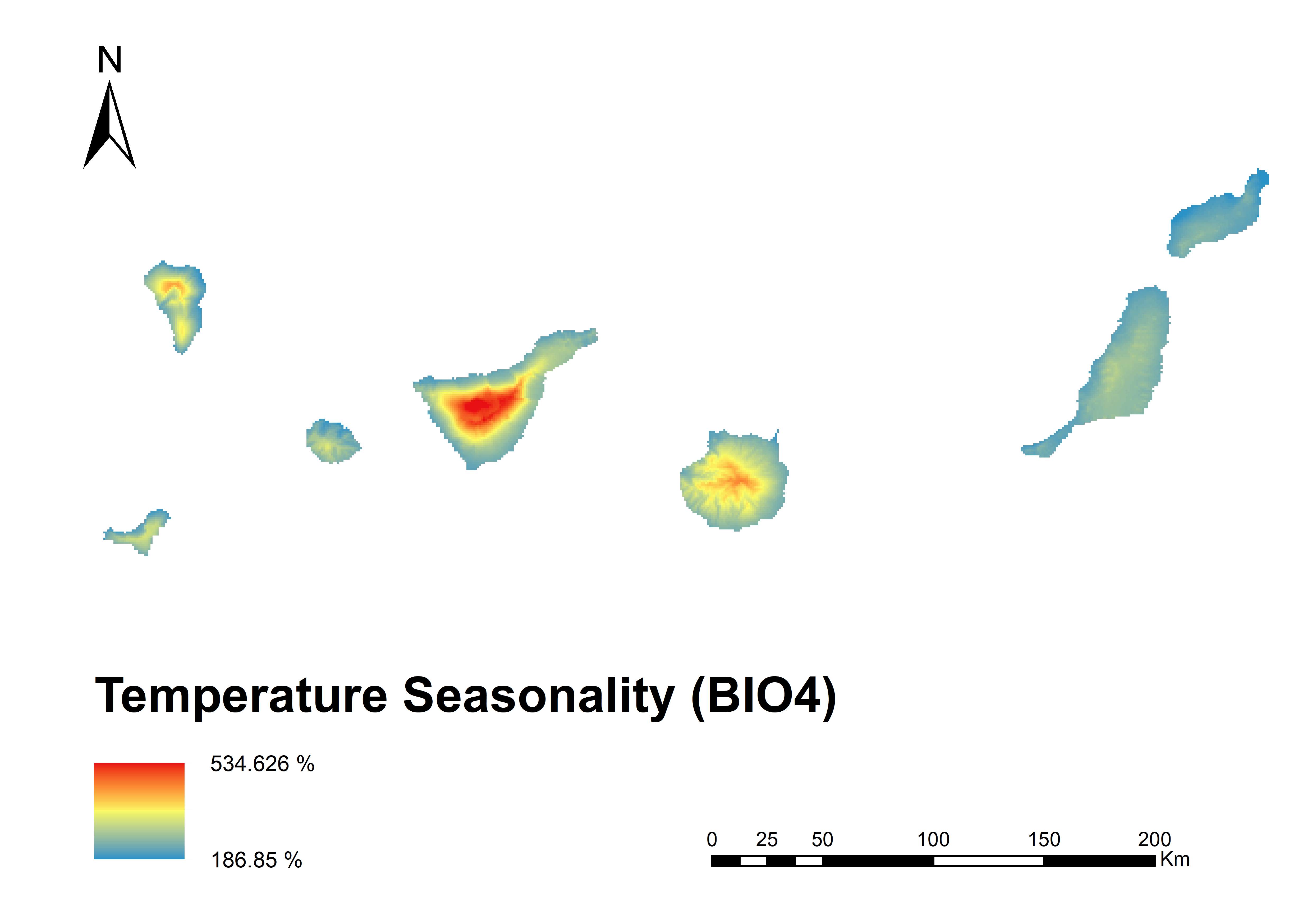

Supplement: Supplementary file 1 [file animals-13-03251-s001.zip › Additional file 3.tif]

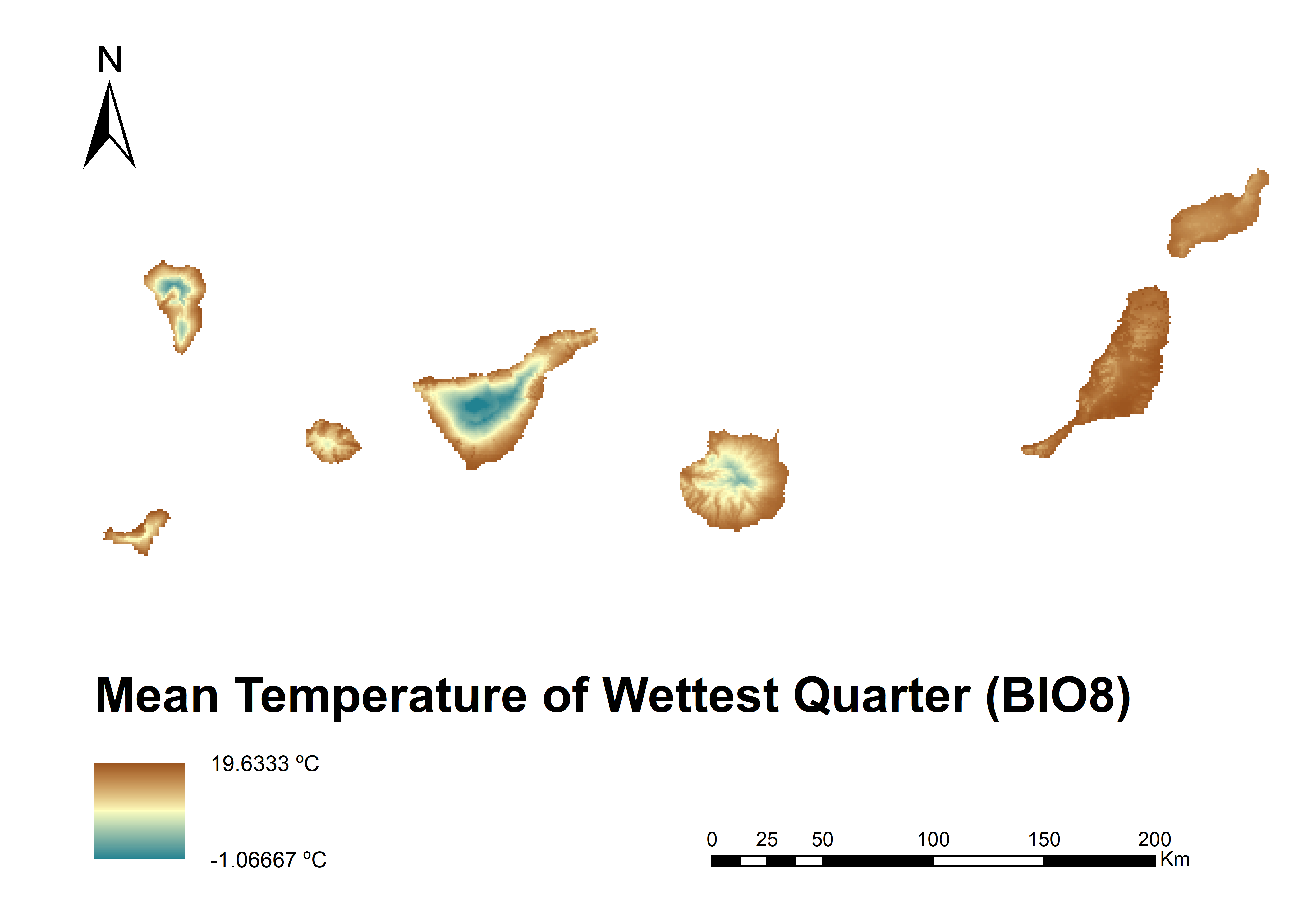

Supplement: Supplementary file 1 [file animals-13-03251-s001.zip › Additional file 4.tif]

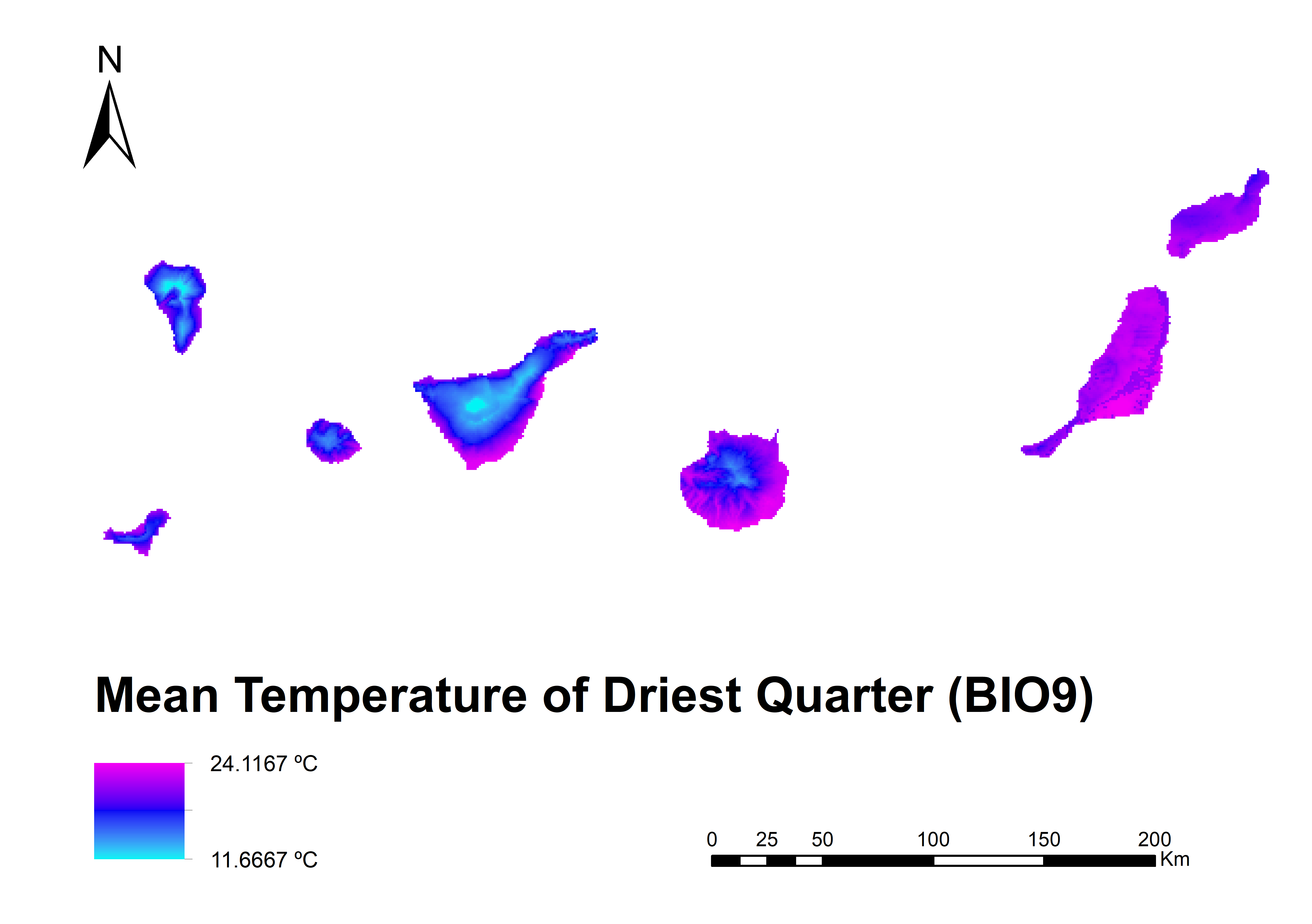

Supplement: Supplementary file 1 [file animals-13-03251-s001.zip › Additional file 5.tif]

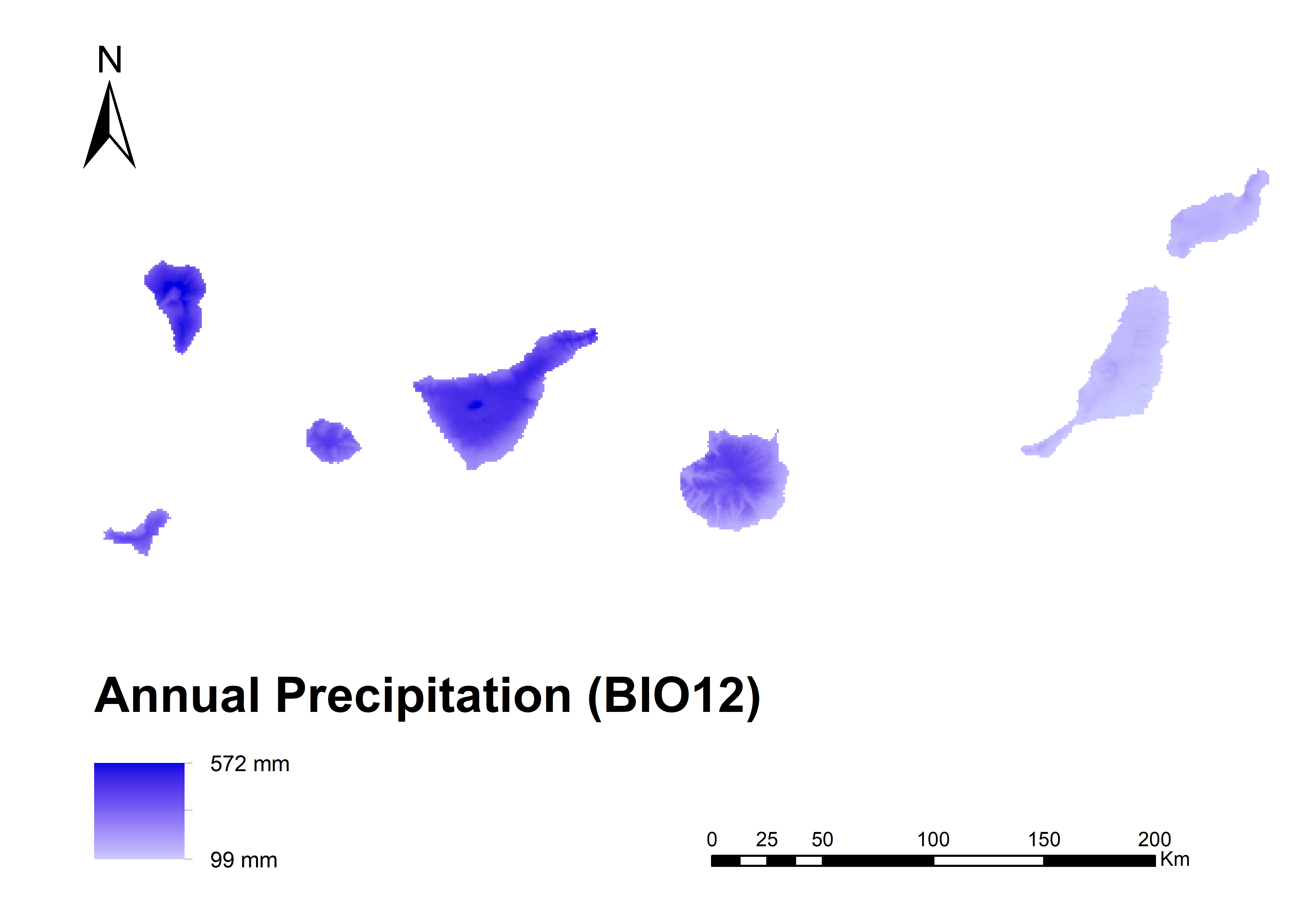

Supplement: Supplementary file 1 [file animals-13-03251-s001.zip › Additional file 6.tif]

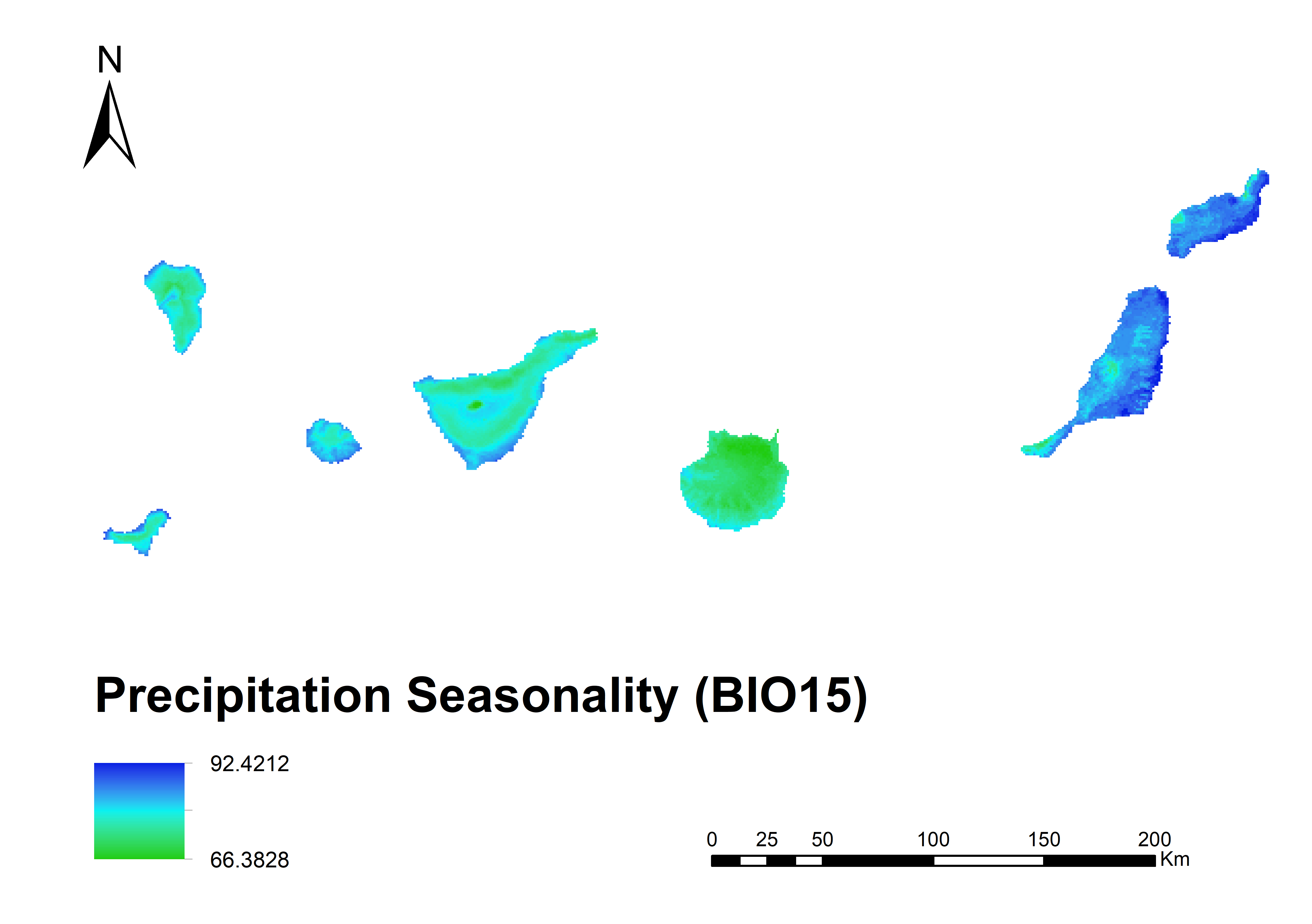

Supplement: Supplementary file 1 [file animals-13-03251-s001.zip › Additional file 7.tif]

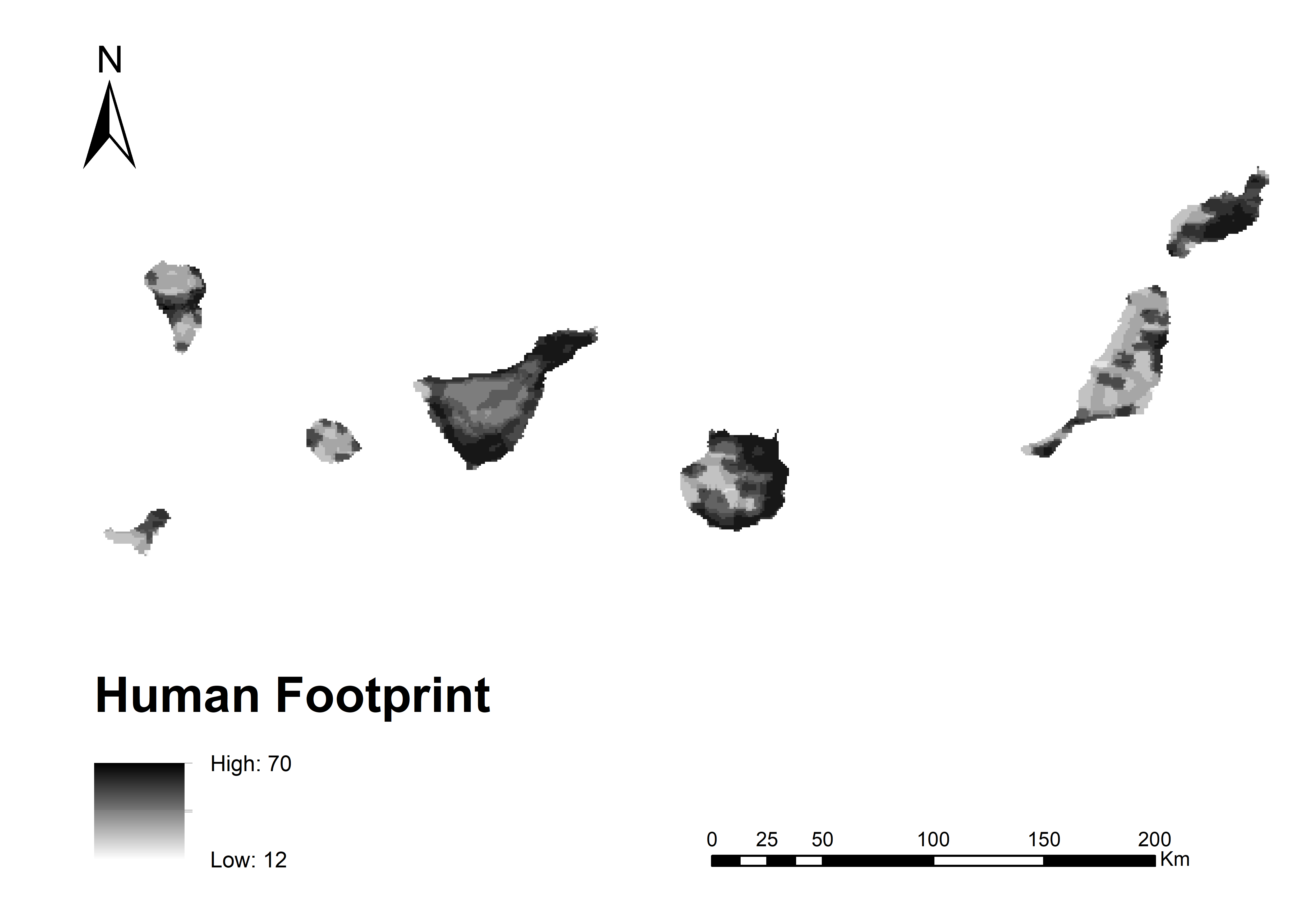

Supplement: Supplementary file 1 [file animals-13-03251-s001.zip › Additional file 8.tif]

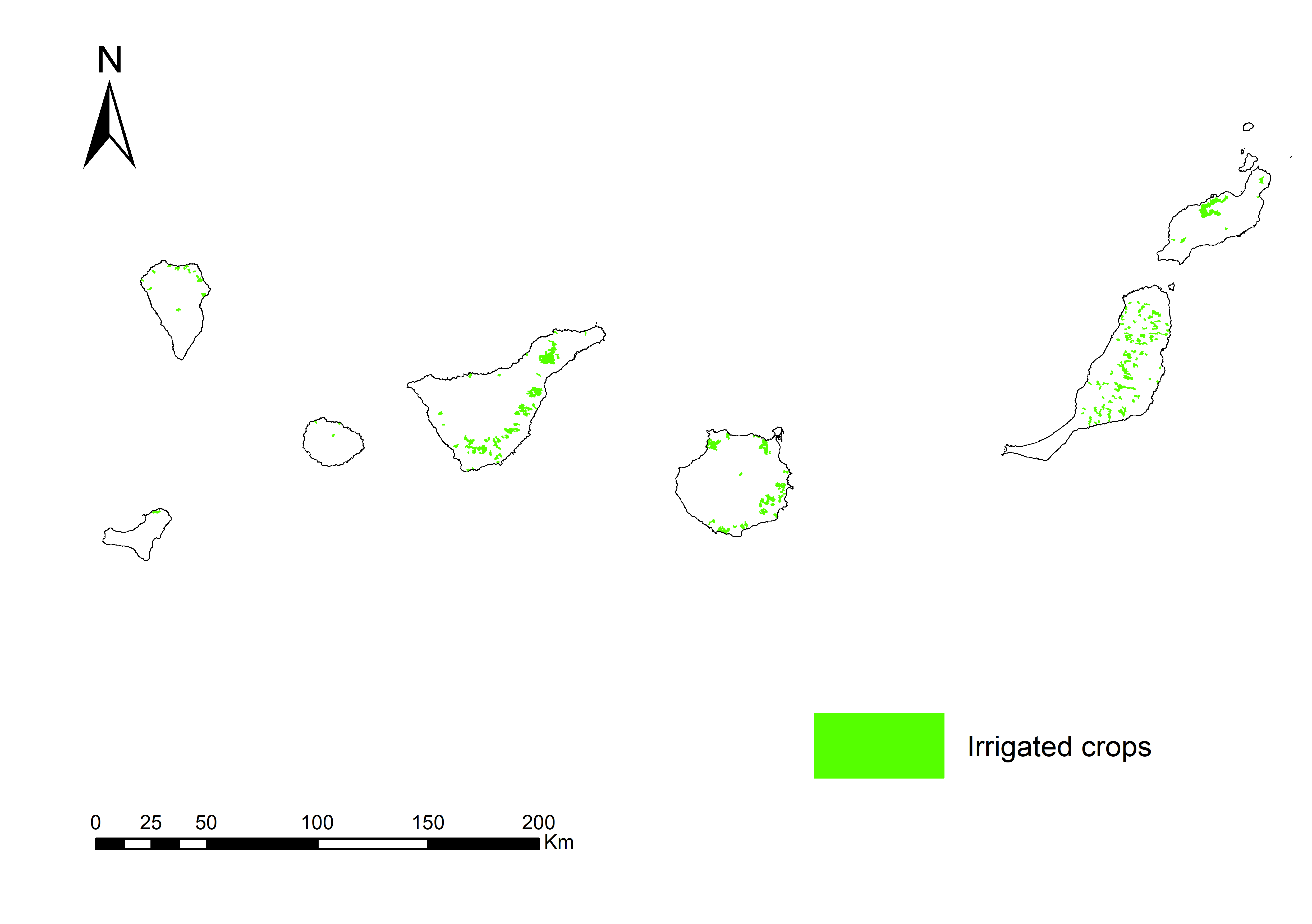

Supplement: Supplementary file 1 [file animals-13-03251-s001.zip › Additional file 9.tif]
